# Anticoccidial Activity of *Aloe debrana* and *Aloe pulcherrima* Leaf Gel against *Eimeria* Oocysts

**DOI:** 10.1155/2020/8524973

**Published:** 2020-05-28

**Authors:** Andualem Yimer Desalegn, Mulubrihan Rahimeto Ahmed

**Affiliations:** School of Veterinary Medicine, Wollo University, P.O. Box 1145, Dessie, Ethiopia

## Abstract

Avian coccidiosis is one of the serious infectious diseases that pose huge impact on the health and production of poultry, hence mainly controlled by regular use of prophylactic and therapeutic chemical drugs. Frequent use of anticoccidial drugs, however, has resulted in the development of resistance in the *Eimeria* species and concerns about drug residues which have stimulated the efforts to search for alternative. *Aloe pulcherrima* and *Aloe debrana* are some of the endemic *Aloe* species of Ethiopia which are traditionally used for the treatment of various infectious diseases. In this study, an *in vitro* trial was undertaken to evaluate the effect of *Aloe debrana* and *A. pulcherrima* leaf gel infusions on the inhibition of the sporulation of oocysts of mixed *Eimeria* species isolated from naturally infected chickens. In this assay, petri dishes containing unsporulated coccidian oocysts at a dose of 1500 oocysts/ml of fecal solution were randomly assigned to 10, 15, 25, and 30% *w*/*v* crude gel infusion of both aloe species in 1% potassium dichromate solution while Amprolium and distilled water served as control groups. The results of this study show that 10, 15, 25, and 3 0% *w*/*v* gel infusions at the tested concentrations have anticoccidial activity as evidenced by their ability to decrease significantly (*P* < 0.05) the sporulation of *Eimeria* oocysts relative to the control incubation. The efficacy of *A. debrana* was found significantly better (*P* < 0.05) than *A. pulcherrima* at different concentrations. However, *A. debrana* at 30% concentration showed significantly higher (*P* < 0.05) sporulation inhibition efficacy of 79.35% (CI: 75.99-83.21) compared to *A. pulcherrima* (69.17%, CI: 64.65-73.92) at similar concentration in relation to the control incubation, though this could not be compared to Amprolium which was more effective (*P* < 0.05) with an inhibition percentage of 90.54% (CI: 89.16-92.21). This study has shown that there is potential for use of *Aloe debrana* leaf gel for the control of avian coccidiosis and as a chemotherapeutic, though much research is needed to determine absolute concentration which will make it comparable to commercially available drugs in terms of efficacy.

## 1. Introduction

Under circumstances of extreme poverty where people cannot keep larger species of livestock due to shortage of land and capital, village chicken provide high-quality animal protein at the source of production and income for household activities [1]. Consequently, poultry production has a major role in the economy of developing countries including an important role in poverty alleviation by means of income generation and household food security, which could especially increase distribution of resources through involvement of women [2–4].

In Ethiopia, the contribution of village chickens to farm household and rural economies is not proportional to their high numbers; the most important constraints are parasitic and infectious diseases and poor housing conditions [5]. Among the infectious diseases of poultry, coccidiosis is one of the commonest and economically most important diseases of poultry worldwide [6]. Poultry coccidiosis in chicken is caused by the intracellular protozoan parasite of Eimeria species in the genus *Eimeria* [7].

In Ethiopia, the disease is of socioeconomic importance among small-scale poultry producers and rural poultry farmers. Poultry coccidiosis importantly become the main constraint for those enterprises and cooperatives that are participated in middle-scale production, which practise intensive and semi-intensive poultry production system in the country. Poultry coccidiosis, caused by *E. acervulina*, *E. necatrix*, *E. maxima*, and *E. tenella*, is endemic and highly prevalent in all parts of the country and affects mainly young growing birds; as a result, it becomes the most important cause of chicken mortalities in all poultry farms in Ethiopia [8].

Coccidiosis causes considerable economic loss in the poultry industry, especially in broiler chicken as it is associated with reduced growth rate and impaired feed conversion thus leading to poor performance of chicken and mortality [9]. The disease is controlled mainly by hygiene and the use of chemical anticoccidial drugs, but mostly, efforts have always been made to add coccidiostates on a regular basis in chicken's diet for the control of coccidiosis [10]. However, the development of drug resistance by the causative parasites and the escalating cost of drug development have greatly reduced the commercial incentive to develop new chemical anticoccidial drugs [11]. As a result, the development of alternative safer and environmentally friendly anticoccidial drugs has become a priority in most parts of the world [12].

Medicinal herbs, as a new class of additives to animal and poultry feeds, have beneficial properties such as antioxidant, antimicrobial, and antifungal [13] as well as immunomodulatory and anticoccidial effects which lead to increased use of herbs. A well-known herb that has received particular attention from researchers is *Aloe*, which is one of the oldest herbs with a history that dates back to traditional medicine thousands of years ago [14]. The gel in *Aloe* leaves contained dry matter which contains more than 75 biologically active ingredients [15] which have medicinal effects that are useful in treating diseases. In this regard, therapeutic properties of *Aloe* have been studied in different animal models and human beings. These include anti-inflammatory, immunomodulatory, wound healing, antibacterial, antiviral, antifungal, antidiabetic, antioxidant, and different enzyme inhibitor activities [16, 17].

Aloe gel is one of the readily available and excessively abundant herbal extracts in Africa that are expected to produce desirable results [18]. Some resource-poor smallholder farmers usually use *Aloe* species to reduce chicken mortalities in Africa. Common examples of *Aloe* used in Southern Africa include *Aloe excelsa*, *Aloe christiana*, and *Aloe spicata* [19]. *Aloe chabaudii* Schonland is also another species of *Aloe* which is endemic in Zimbabwe and Mozambique which also has been used to control avian coccidiosis [20].

About 46 species of *Aloe* are known so far in Ethiopia of which about 24 of aloe species are endemic to the country [21]. *Aloe pulcherrima* and *Aloe debrana* are some of the endemic aloe species of Ethiopia which are traditionally used medicinally by herbalists for the treatment of various infectious diseases in central and northern part of Ethiopia [22].

*Aloe debrana* is an endemic species in Shewa, Gojam, and Wollo in Amhara Region, north central Ethiopia [23]. According to Demissew and Nordal [24], *Aloe debrana* is restricted in southwest of Debre Berhan town on the road to Dessie town Amhara Region with main flowering period in the dry season [25].

*Aloe pulcherrima* is recognized by the pale blue-green leaves with fine distinct longitudinal lines and lacking marginal teeth. The species is endemic in Gonder, Gojam, Wollo and Shewa floristic regions of Amhara Region, north central Ethiopia [25]. The antibacterial and antimalarial effects of both aloe species were studied by different researchers in Ethiopia: *Aloe pulcherrima* leaf by Tekleab et al. [26] and Abdissa et al. [27]; similarly, *A. debrana* leaf extracts were studied and reported by Tekalign et al. [28]. However, unlike its former mentioned *Aloe* species studied in western Africa, there is not much evidence in terms of quantifiable data to be used regarding our endemic *Aloe* species as anticoccidial agents in Ethiopia.

Recently, a number of in vitro experiments have proved remarkable anticoccidial effects of different herbal extracts and essential oils on inhibition of sporulation of coccidian oocysts. These results suggest herbal anticoccidials open new perspective and proved suitable to serve as alternative to conventional treatment particularly in countries with limited economic potential [29]. Therefore, the present study was conducted to evaluate in vitro sporulation inhibition efficiency of different concentrations of *Aloe pulcherrima* and *Aloe debrana* leaf gel infusions against Eimeria oocysts.

## 2. Material and Method

### 2.1. Description and Selection of Study Area

*Aloe pulcherrima* and *Aloe debrana* were the candidate medicinal plants for this study, and the study was conducted from December 2016 to November 2017. Selection of the two aloe species as candidate medicinal plants for this study was based on their abundant availability in the study area, and also, their leaf has high gel content [21]. The candidate aloe species were collected from Dessie Zuria district of South Wollo Zone, Amhara Region, Northeast Ethiopia, and is found at 401 km from Addis Ababa. The selection of the two PAs was depending on the dominant availability of *Aloe debrana* and *Aloe pulcherrima* in the particular area after conducting survey. Fresh aloe leaves of *Aloe pulcherrima* were collected from “Tita” mountain, Dessie Zuria district, and *Aloe debrana* leaves were collected from wild aloe plants of Harawobelo PA (5 km from Dessie town towards Addis Ababa, the capital city of Ethiopia). The two aloe species were collected from the specific area during their flowering month of the study period.

### 2.2. Aloe Species Identification and Collection

The plant collection was conducted in two phases, and sampling method was purposive. In the first phase, the survey was undertaken to identify the *Aloe* species based on their physical characteristics (colour, leaf arrangement, height, edge spot, stem, and flower) according to the identification criteria (morphological characteristics combined with geographical distribution) of *Aloe* species of Ethiopia as described by [23–25]. In the second phase, the identified aloe species were collected from the selected areas. The aloe species were identified and confirmed as *Aloe pulcherrima* and *Aloe debrana* by experts of botanical sciences in the biology department of Wollo University. After the spices are identified and confirmed, ten (10) *Aloe pulcherrima* and *Aloe debrana* leaves (two leaves per plant) were collected from randomly selected different wild plants and stored overnight in a refrigerator at a temperature of 4°C to prevent oxidation of a heat-degradable component.

### 2.3. Description of Research Procedures and Instruments

As aloe gel has been reported to contain compounds with anticoccidial [18, 30], antioxidant [31], and immunostimulatory [18, 32] effects against coccidiosis in chickens, due to these properties, we prefer the gel part of the candidate aloe species as anticoccidial remedy for this in vitro experiment and for further in vivo tests. Furthermore, aloe gel contains several beneficial ingredients including vitamins, minerals, enzymes, organic acids, and carbohydrates which could improve production performance of chicken in addition to its anticoccidial effects [33]. Another criteria to use the gel part of aloe leaf as anticoccidial agent in this study were due to the efficacy and safety of aloe gel in the treatment of inflammation of the gastrointestinal tract. According to Langmead et al. [34], a higher dose of *Aloe vera* gel has been found more efficacious in the treatment of active ulcerative colitis. However, aloe latex efficacy is strongest for the laxative effects for treatment of constipation; moreover, the yellow sap in the latex has bitter taste and is associated with considerable toxicity risks.

#### 2.3.1. Crude Gel Extraction and Preparation of Different Concentrations of Gel Infusions

Thorns on the aloe leaves were removed using a scalpel blade, and the leaves were thoroughly washed using water before preparation for extracting juice after which latex of the leaf was removed from the leaf manually by making a cut using a pocket knife and crude gel was collected in different beakers from each species. The crude gel was thoroughly mixed to obtain uniform solution using an electric blender and then stored in sample bottles. The gelatinous material obtained was filtered through a funnel to remove the floating material in the suspension. The aloe gel extracts were stored in a refrigerator at 4°C prior to use to avoid oxidation, thereby maintaining the active ingredients in the gel extracts.

The extracted gel was reconstituted in distilled water at 10, 15, 25, and 30% (*w*/*v*) concentrated infusions which were prepared by taking 10-, 15-, 25-, and 30-gram mass of crude gel in a glass bottle from both candidate aloe species, and 100 ml of sterile distilled water was poured on it. Then, the bottles were shaken for 5-7 minutes to ensure through mixing and were then kept for 6-8 hours at room temperature prior to use [35]. The test concentrations (10, 15, 25, and 30%) of the aloe leaf gel in this study were selected based on the recommendation of the WHO [36] for the posology of fresh aloe gel, which is a preparation containing 10-70% fresh gel concentration.

#### 2.3.2. Collection of Eimeria Oocysts

The *Eimeria* oocysts used in this study were derived from a rural chicken suffering from natural clinical coccidiosis from Kombolcha veterinary clinic in South Wollo, Ethiopia. Following evisceration at postmortem, the caeca and intestines were separated and sliced open longitudinally and their contents were washed into a beaker using tap water. The washed contents were centrifuged, and the sediment remixed with saturated sodium chlorine solution (NaCl) to make the oocysts float [37]. The collected oocysts were allowed to sporulate at room temperature in 2.5% potassium dichromate solution. The harvested oocysts were multiplied in 6 chicks following oral infection which are to be used as source of *Eimeria* oocysts for purposeful infection (used as positive reservoir hosts of *Eimeria* oocysts). The chicks were routinely monitored daily for the development of clinical coccidiosis and the presence of *Eimeria* oocysts in their feces. The fecal oocyst counts were determined from the mixed fecal sample by the modified McMaster technique to determine the number of oocysts per gram of the fecal sample [10]. Different species of genus *Eimeria* were identified based upon their morphology and size according to the standard identification keys [38, 39], and the proportion of different *Eimeria* species isolated was assessed. The collected species of *Eimeria* oocysts were kept at the Parasitology Laboratory, School of Veterinary Medicine, Wollo University until their use for this *in vitro* study.

#### 2.3.3. Experimental Design and Sporulation Inhibition Assay

In this experimental study, an in vitro sporulation inhibition assay was used to evaluate the effect of different concentrations of *Aloe debrana* and *Aloe pulcherrima* gel infusions on the sporulation of coccidian oocyst. About 10 g of fecal samples collected from reservoir chickens infected with mixed species of *Eimeria* oocysts was taken to Wollo Veterinary Parasitology Laboratory for oocysts counting using the McMaster chamber oocyst counting technique. The samples were brought to the laboratory in a 50 ml centrifuge tubes containing 2% (*w*/*v*) potassium dichromate to prevent bacterial degradation of oocysts during transportation. The samples were processed as described by Taylor et al. [40] with some modifications. Briefly, 3 g of fecal sample was transferred from the centrifuge tube into a glass beaker and mixed with 1 ml of 2% (*w*/*v*) potassium dichromate before adding 41 ml of water. The mixture was thoroughly mixed for 1 min, and the resulting suspension was strained through double-layered cheesecloth into a beaker. The filtrate was thoroughly mixed before transferring 15 ml to a centrifuge tube and then centrifuged for 5 min at 1500 rpm. After removing the supernatant, the pellet that remained in the test tube was resuspend with 15 ml saturated flotation salt solution. After mixing well for 1 min, 1.5 ml of samples was taken from the solution using a disposable plastic pipette and was poured on a double-chambered McMaster slide. The tube was mixed again before the second chamber of the McMaster slide was filled with a subsample. The preparation was left for 5 min before counting, allowing the oocysts to float up. All the oocysts under the grid of each chamber in the McMaster slide were counted using ×10 objective, and the mean of the two counts was calculated. This process was repeated twice, and a count of one oocyst was equivalent to 300 (45 ml—the total volume of the original suspension of feces/0.15 ml—the volume of the sample examined) oocysts/sample [40, 41].

#### 2.3.4. Sample Preparation for Incubation and Counting

The prepared oocyst potassium dichromate water mixture was centrifuged for 5 minutes at a gravity of 2000 rpm so as to increase oocyst counts in 1 ml oocyst water potassium dichromate mixture/solution. The supernatant was discarded until 5 ml of solution was left. The number of unsporulated oocysts in the original aqueous suspension of feces was adjusted to 1500 oocysts per ml.

Separate petri dishes containing 10 ml of 10, 15, 25, and 30% *w*/*v* concentration of both *Aloe* species crude gel, 0.125% Amprolium, and distilled water as control group were placed in separate petri dishes labeled appropriately. Amprolium was used for comparison at the recommended concentration (1.25 g/liter) as reference drug, and 1 ml fecal suspension with equal number of oocysts (1500 oocyst/ml) was withdrawn using a 5 ml Pasteur pipette and randomly allocated to each of the prepared petri dishes into which various aloe leaf gel infusions (10, 15, 25, and 30% *w*/*v*) for each candidate aloe species, 0.125% Amprolium, and the control group were then mixed properly. Three replications were made for each concentration of the different aloe species leaf gel infusions. In this assay, the unsporulated oocysts in all treatment groups were incubated for 48 h at 29°C using water baths to maintain constant temperature as per Conway and McKenzie [39]. At the end of the incubation, the sporulated oocysts were washed twice in tap water and stored at 4°C until being counted. The average count of two subsamples was considered for the total number of sporulated and unsporulated oocysts of suspension of each treatment. The inhibition efficacy was measured in percentage, and the number of sporulated and nonsporulated oocysts was counted, and the percent sporulation was estimated by counting the number of sporulated oocysts in a total of 100 oocysts. The oocysts with 4 sporocysts were considered sporulated regardless of the shape and size of the sporocysts [42].

### 2.4. Statistical Analysis

The data collected were entered and analyzed using SPSS, version 20. The sporulation inhibition efficacy was measured in percentage which represented the *in vitro* proportion of sporulated and unsporulated oocysts [42]. Data are presented as the mean ± SEM, and one-way analysis of variance (ANOVA) followed by Duncan's multiple range test was employed for detection of significance among treatment groups. *P* < 0.05 was considered statistically significant.

## 3. Results

The present study was planned to evaluate an *in vitro* efficacy of different concentrations of two endemic aloe species, which are abundant and easily available in the study area, on mixed Eimeria oocysts. Mixture of the oocysts used for this sporulation inhibition assay was isolated and consists of *E. tenella* 35%, *E. acervulina* 27%, *E. necatrix* 20, and *E. maxima* 18% with an average fecal oocyst count of 2600 oocysts per gram of fecal sample (Table 1). The present in vitro study revealed that both aloe species induced anticoccidial effect which was concentration dependent and increased by increasing the concentration of the tested aloe crude gel infusions.

Different concentrations from *Aloe debrana and Aloe pulcherrima* crude gel infusions at concentrations of 10, 15, 25, and 30%, were prepared. For comparison, 0.125% Amprolium was used at the recommended dose of 1.25 g/liter as reference drug. Separate petri dishes containing 10 ml of each concentration of gel of both plants were inoculated with equal number of viable oocysts (1500 oocysts/1 ml) of fecal suspension. The mean percentage inhibition of spoliation tested of aloe gel was recorded after 72 hr. postexposure accordingly; different concentrations of aloe gel showed concentration-dependent inhibition for the sporulation of coccidial oocysts of mixed *Eimeria* species as compared to the control group (K2Cr2O7 and 1500 oocysts/1 ml), as shown in Tables 2 and 3.

According to this study, only about 12.42% (95% CI: 8.42-23.65) of the oocysts of *Eimeria* were found inhibited from sporulation in the control incubations containing oocysts and distilled water whereas incubations containing 10%, 15%, 25%, and 30% *Aloe debrana* gel infusions showed 55.72% (95% CI: 50.64-60.45), 61.48% (95% CI: 58.9-63.45), 71.67% (95% CI: 68.61-75.44), and 79.35% (95% CI: 75.99-83.21) oocyst sporulation inhibition efficacy, respectively, relative to control incubations, while the treatment group of Amprolium at 1.25 g/l was found to show 90.54% (95% CI: 89.16-92.21) inhibition in sporulation efficacy relative to the control treatment incubation (Table 2). Accordingly, the sporulation inhibition percentage was found to be increased when the concentration of *Aloe debrana* gel is increased (Table 2).

The statistical analysis showed that all concentrations of the tested Aloe species gel significantly inhibited the sporulation of *Eimeria* oocyst as compared to the control group (*P* < 0.05); however, there was no significant difference (*P* > 0.05) among 10% and 15% *Aloe debrana* leaf gel concentrations in sporulation inhibition efficacy, reflecting that increasing the concentration until 15% did not significantly inhibit oocysts from sporulation. This finding showed that use of *Aloe debrana* gel infusion above 25% concentrations significantly increase sporulation inhibition of *Eimeria* oocysts (Table 2).

The current sporulation inhibition efficacy test revealed that *Aloe pulcherrima* gel treatment at concentrations of 10, 15, 25, and 30% *w*/*v* resulted in 46.1% (95% CI: 44.03-48.45), 50.32% (CI: 43.88-56.38), 53.5 (CI: 42.31-56.38), and 69.3% (CI: 64.65-73.92) reduction in sporulation (inhibition of sporulation) relative to the control treatment incubation, while the treatment group of Amprolium at 1.25 g/l was found to have 88.75% (95% CI: 85.56-90.21) inhibition in sporulation efficacy relative to the control treatment incubation (Table 3).

Generally, in this result, both the studied aloe species *A. debrana* and *A. pulcherrima* were significantly different in inhibiting sporulation of coccidian oocysts (*P* < 0.05) at different gel concentrations. The increase in aloe concentration had an effect on the sporulation inhibition of coccidian oocysts (*P* < 0.05); the percentage of unsporulated oocysts increased with increasing aloe treatment concentrations (Tables 2 and 3). The 10 and 15% *w*/*v* of *A. debrana* concentrations were not significantly different in inhibiting coccidian oocyst sporulation, while the 10% *A. pulcherrima* concentration was not different from 15% and 25% *A. pulcherrima* concentrations. Amprolium at 1.25 g/l dose® treatment had the highest percentage of unsporulated oocysts in comparison to aloe gel treatments; however, the maximum concentration (30%) of both aloe gels was found significantly (*P* < 0.05) lower than that of the Amprolium treatment in inhibiting sporulation of oocysts (Tables 2 and 3). *A. debrana* leaf gel inhibited sporulation better than *A. pulcherrima* at all the tested treatment concentrations of both species.

## 4. Discussion

Coccidiosis is an economic and health problem in the poultry industry and can infect any type of poultry in any type of facility, and its occurrence is worldwide [43]. In the recent years, botanicals have got great attention for the control and treatment of infectious diseases of animals [44, 45]. Several poultry scientists all over the world are now actively engaged in research into the use of plants and plant-derived products to fight and reduce the heavy economic losses in poultry industry caused by coccidiosis [46]. A number of botanicals having anticoccidial potential like *Saccharum officinarum* [47], *Pinus radiata* [48, 49], and *Aloe vera* [50, 51] with promising anticoccidial effects have been reported in different researches.

Sporulation inhibition test is a common criterion to assess the anticoccidial properties of botanicals as alternative to synthetic anticoccidials [49], based on the effects of some botanicals on the sporulation inhibition of coccidian oocysts. Molan and Thomas [52] studied the effects of aqueous extracts from green tea on the sporulation of *E. tenella*, *E. acervulina*, and *E. maxima*; accordingly, unsporulated oocysts incubated with 10% and 25% (*v*/*v*) green tea extracts were found to significantly reduce the sporulation rate of the oocysts. Similar in vitro inhibition assay results by Molan et al. [49] showed that aqueous extracts of pine bark have potential to inhibit sporulation of *Eimeria* oocysts.

The findings of the present study indicate that different concentrations of crude aloe gel of the two studied endemic aloe species (*Aloe debrana* and *Aloe pulcherrima*) have anticoccidial activity as evidenced by their ability to decrease significantly the sporulation of unsporulated *Eimeria* oocysts (*P* < 0.05) compared with the control incubation. This observation showed that aloe gel infusions are able to kill or inhibit the growth and development of oocysts to become the infective stage of *Eimeria* (sporozoites). The finding that *A. debrana* at 30% (*w*/*v*) concentration had significantly (*P* < 0.05) the least sporulation proportion compared to *A. pulcherrima*at similar concentration could suggest that *A. debrana* was most effective in killing *Eimeria* oocysts with a sporulation inhibition efficacy of 79.35% (95% CI: 75.99-83.21), while *A. pulcherrima* was found to have a 69.17% (95% CI: 64.65-73.92) inhibition efficacy.

In agreement with the current findings, similar in vitro effects of *Aloe vera* and *Aloe spicata* on the inhibition of the sporulation of avian coccidian oocysts were reported by Mwale et al. [53] and they found that *Aloe spicata* had higher sporulation inhibition efficacy at 30% concentration (65.42%) while *A. vera* had the least number of sporulated oocyst at 45% concentration with an inhibition percentage of 62.5%. The 79.35% inhibition efficacy of *A. debrana* at 30% (*w*/*v*) concentration found in this study is consistent with the findings by El-Khtam et al. [54] who reported 80% sporulation inhibition effects of garlic powder. Furthermore, El-Khtam et al. [54] reported 66% inhibition efficacy of turmeric powder which is comparable to the 69.3% inhibition efficacy of *A. pulcherrima* at 30% concentration found in this study.

Many compounds with diverse structures have been isolated from both the central parenchyma tissue of *A. vera* leaves (the gel) and the exudate (the latex). The bitter yellow exudates which are known as the latex contain 1,8-dihydroxyanthraquinone derivatives and their glycosides which are mainly used for their cathartic effects [55]. The aloe parenchyma tissue or the gel has been shown to contain proteins, lipids, amino acids, vitamins, enzymes, inorganic compounds, and small organic compounds in addition to the different carbohydrates, with reports of chemotaxonomic variation in the polysaccharide composition of different aloe species [56]. According to the evaluation report of cosmetic ingredient review expert panel (CIR) [57], the polysaccharide material derived from the inner gel is noncytotoxic. However, the whole leaf extract and latex of aloes were found to be cytotoxic, mutagenic, and carcinogenic due to the anthraquinones and other phenolic compounds found in whole leaf and latex of aloes [15, 58].

Sherafatmanesh and Ekramzaden [59] reported that *Aloe vera* is one of the most well-known herbal medicine with considerable properties such as hypolipidemic, hypoglycemic, anticancer, antioxidant, anti-inflammatory, immunomodulatory, wound healing, antibacterial, and antiviral activities. Many of the health benefits associated with *Aloe vera* have been attributed to its various bioactive natural components such as vitamins, anthraquinones, and polysaccharides. There are also several scientific studies supporting the antimicrobial and antioxidant activities of substances found in *Aloe* leaf gel; these biological activities of the gel are due to the synergetic action of compounds found in aloe gel. Mainly the polysaccharides in *A. vera* gel have therapeutic properties such as immunostimulation, anti-inflammatory effects, wound healing, antibacterial, antiviral, antifungal, stimulation of hematopoiesis, and antioxidant effects [60, 61]. The polysaccharides particularly mannose-containing polysaccharides, cellulose, and pectic polysaccharides comprise the major part of *Aloe vera* gel. According to Hamman [62], acetylated glucomannan is the primarily responsible component for the gel's mucilaginous properties and has been found to modulate immune function and accelerate wound healing [63]. Furthermore, the anti-inflammatory potential of *Aloe vera* gel has been demonstrated in vitro by Esua and Rauwald [64] which showed that maloyl glucans isolated from *Aloe vera* gel have potent anti-inflammatory effects as well as antagonistic effects on cell proliferation. On the other hand, certain results showed that among the nonpolysaccharide *Aloe vera* gel constituents, salicylic acid and other antiprostaglandin compounds may contribute to the local anti-inflammatory activity of *Aloe vera* gel by inhibition of cyclooxygenase to reduce pain, inflammation, and oedema in areas of inflammatory lesions [63]. Furthermore, the antioxidant potential of the extracts of Aloe vera (leaf and flower) has been reported by Lopez et al. [65]. Similarly, Hamman [62] showed that *Aloe vera* has a dose-dependent antioxidant effect which is helpful in the treatment of various diseases. Particularly, Ulbricht et al. [63] reported that potent antioxidant effects, including the ability to scavenge superoxide anions, have been attributed due to the effects of aloes in a derivative found in the gel of aloe.

Furthermore, variation in the distribution of typical aloe chemical components in whole leaf extract and root was found different between endemic aloe species in Ethiopia [66]. According to Dagne et al. [66], *A. pulcherrima* revealed the conspicuous absence of a chemical isolate known as barbaloin in its leaf extract, but this component was isolated from *Aloe debrana* as one of its major components. However, *A. pulcherrima* uniquely contains nataloin and 7-hydroxyaloin as its major isolate constituents [66]. Considering the variation in major photochemical compounds in different endemic aloe species, previous *in vivo* and *in vitro* studies by different researchers in Ethiopia have shown *Aloe* species possessing strong antimalarial activity. An in vivo antiplasmodial activity test of methanolic leaf extract of *A. debrana* showed a dose-dependent chemosuppressive effect [28]. Similar studies on the antimicrobial effects of the leaf latex [67] and the roots of *A. pulcherrima* have been shown to have antibacterial [67 and 8], antifungal [67], and antiplasmodial [8] activities. A recent in vitro study on the rhizome of *A. pulcherrima* has shown the highest antibacterial and antiplasmodial activity against chloroquine-resistant (W2) strain of *Plasmodium falciparum* [68].

The significant difference (*P* < 0.05) between the two aloe species in inhibiting sporulation of the *Eimeria* oocysts at similar concentrations in the current study may be due to difference in chemical compositions of the studied aloe species. This is supported by the report of Yim et al. [69], which showed that *Aloe vera* treatment was found successful in the control of coccidiosis and its efficacy is due to the chemistry of its gel.

Sacan et al. [70] conclude that *A. vera* leaf gel extract could be used as a source of natural enzyme inhibitor and antioxidant activity in pharmaceutical, cosmetic, and food industries. According to El-Khtam et al. [54], antioxidant-rich plants may be lethal to the parasites by inducing oxidative stress and neutralize reactive oxygen species and have potential benefits in treating coccidial infections.

It can be speculated that the antioxidant phytochemicals of the polysaccharide derivatives, in aloe gel, exhibited antisporulation effect by interfering in the physiological process necessary for sporulation process like preventing access of oxygen (inhibition of oxygen consumption of the cells) and inhibition of various enzymes responsible for sporulation as reported in helminth eggs by Zaman et al. [71]. The anticoccidial effect of aloe gel may be attributed to its anticoccidial property and its saponin content, which act on the protozoan development by interacting with cholesterol present on the parasitic cell membrane and resulting into parasitic death [71, 72], while its glutathione peroxidase activity, superoxide dismutase enzymes, and a phenolic antioxidant were found to be present in *A. vera* gel which may be responsible for these antioxidant effects [73]. According to the in vitro test results of Langmead et al. [73], *A. vera* gel has a dose-dependent antioxidant effect. Since this study was the first trial on anticoccidial efficacy of endemic aloe species in Ethiopia, there is no information on the photochemical analysis and antimicrobial mechanism of these studied aloe species particularly their leaf gel; however, the effect of these tested aloe species may be attributed to their antioxidant effects as a major application on sporulation inhibition.

This result also showed that the most effective aloe species *A. debrana* at 30% *w*/*v* concentration although effective had a significantly lower sporulation inhibition value as compared to the commercially available anticoccidial drug Amprolium at a dose of 1.25 g/liter of water with a sporulation inhibition efficacy of 90.54%. Other similar in vitro sporulation inhibition test in Egypt showed that garlic powder at a concentration of 5 g/liter of water with 80% efficacy was lower than Amprolium with an inhibition efficacy of 90% [54], which is exactly similar with the recorded maximum inhibition efficacy of Amprolium (90.54%, 95% CI: 89.16-92.21) in this study (Table 2). The current finding is also in agreement with the report by Sungirai [35], where *A. chabaudii* gel was not as efficient in its oocyst reduction as compared to the commercial sulphonamide (ESB3). The most possible reason for this could be that the concentration levels of the studied aloe gel in this experiment were not enough to conclude that *A. debrana* leaf gel could not be as effective as Amprolium in sporulation inhibition efficacy. This implies that the sporulation inhibition efficacy of the two studied aloe species significantly related with increase in gel concentration was not exhausted to the threshold level. Consequently, further increasing the aloe concentration could possibly have increased the sporulation inhibition efficacy of the tested aloe leaf gel.

Another possible reason for aloe treatments in this study which were not found to be as effective as Amprolium may be related with the difference in the mode of action to inhibit sporulation of oocysts between the two agents. Amprolium is a coccidiocidal synthetic drug which kills different stages of oocysts by inhibiting protein synthesis within their systems and thus killing the microbes. This result also showed that though Amprolium was effective, however, it could not be able to inhibit sporulation of Eimeria oocysts to zero. This could be explained by the issue of emergence of drug-resistant *Eimeria* species due to the continuous use of Amprolium to control coccidiosis in the study area. As explained by [74], there has been a concern in the rise of ionophore and chemical drug-resistant strains of coccidian in many poultry production systems of the world and much research is being done to investigate such problems. Similarly, different researches indicated the emerging problems with increasing resistance to *Eimeria* species and concerns about drug residues have stimulated the efforts to search for alternative control measures [75–77].

## 5. Conclusion

This study demonstrated the inhibitory potential of *Aloe debrana* and *A. pulcherrima* on sporulation of coccidian oocysts, *A. debrana* leaf gel inhibited sporulation of *Eimeria* oocysts at significantly higher inhibitory efficacy than *A. pulcherrima* gel at the tested concentrations. However, *A. debrana* is not as effective as Amprolium in inhibiting the sporulation of oocysts; therefore, further studies need to be undertaken to evaluate and establish the optimum concentration of *A. debrana* that should be used to control coccidiosis. Results of the present study suggest to undertake further in vivo research to validate the effect of aloe to provide an organic anticoccidial alternative for the control of coccidiosis. Further chemical analyses are recommended on the two studied endemic aloe species to identify the components responsible for anticoccidial activities.

## Figures and Tables

**Table 1:** Oocyst count and proportion of different *Eimeria* species isolated from naturally infected chickens in the study area.

Isolated *Eimeria* species	Percent (%)	Oocyst/g feces
*E. tenella*	35	
*E. acervulina*	27	
*E. necatrix*	20	
*E. maxima*	18	
Total	100	2600

**Table 2:** Effect of *Aloe debrana* on percentage inhibition of *Eimeria* oocyst sporulation at different concentrations of the leaf gel infusions.

Treatment groups	Number of oocyst/12 ml	Inhibition percentage (unsporulated oocyst) (%)	95% CI
AD-10%	1500	55.72^a^	50.64-60.45
AD-15%	1500	61.48^a^	58.90-63.45
AD-25%	1500	71.67^b^	68.61-75.44
AD-30%	1500	79.35^ab^	75.99-83.21
Amprolium (1.25 g/l)	1500	90.54^c^	89.16-92.21
Control	1500	12.42^d^	8.42-23.65

^a, b, c^Values in a column followed by the same superscripts are not significantly different (*P* > 0.05) while different superscripts indicate statistical difference (*P* < 0.05); AD: *Aloe debrana*.

**Table 3:** Effect of *Aloe pulcherrima* on percentage inhibition of *Eimeria* oocyst sporulation at different concentrations of the leaf gel infusions.

Treatment groups	Number of oocyst/12 ml	Inhibition percentage (unsporulated oocyst) (%)	95% CI
AP-10%	1500	45.52^a^	44.03-48.45
AP-15%	1500	52.32^a^	43.88-56.38
AP-25%	1500	58.8^a^	42.31-64.84
AP-30%	1500	69.17^b^	64.65-73.92
Amprolium (1.25 g/l)	1500	88.75^ab^	85.56-90.21
Control group	1500	11.38^c^	

^a, b, c^Values in a column followed by the same superscripts are not significantly different (*P* > 0.05) while different superscripts indicate statistical difference (*P* < 0.05); AP: *Aloe pulcherrima*.

## Data Availability

The data used to support the findings of this study are available from the corresponding author upon request.
